# Supplementary material for: Genetic diversity and molecular evolution of Ornithogalum mosaic virus based on the coat protein gene sequence
Source: PeerJ. 2018 Mar 27;6:e4550. doi: 10.7717/peerj.4550 (PMC5877448; doi:10.7717/peerj.4550)
Supplement: Table S1 [file peerj-06-4550-s001.pdf]

Table S1 OrMV isolates used in this study

Isolate	Country	Host origin (Genus)	Collection_date	ACCESSION
C7	Japan	Ornithogalum	8-Feb-02	AB079647
C22	Japan	Ornithogalum	n/a	AB079648
C23	Japan	Ornithogalum	n/a	AB079649
C28	Japan	Ornithogalum	n/a	AB079650
7-3	Japan	Ornithogalum	19-Sep-02	AB091835
G	Japan	Ornithogalum	n/a	AB091836
OMV-IL	Israel	Chenopodium	28-Jul-98	AF080587
Au-Iris	Australia	Iris	11-Nov-99	AF203528
Hangzhou	China	Narcissus	Jan-00	AJ493580
VM-19	India	Vanilla	n/a	AY845013
VM-33	India	Vanilla	n/a	AY845014
nzOrMV-1	New Zealand	Ornithogalum	n/a	AY994102
nzOrMV-2	New Zealand	Iris	n/a	AY994106
nzOrMV-3	New Zealand	Iris	n/a	AY994107
OMV-O	South Africa	Ornithogalum	1990	D00615
Lac33	South Africa	Lachenalia	2007	FJ159371
LachX	USA	Lachenalia	2007	FJ159372
LQD2	USA	Lachenalia	2006	FJ159373
OSS-1	USA	Ornithogalum	2006	FJ159374
OSS-5	USA	Ornithogalum	2006	FJ159375
Gla-11	Netherlands	Gladiolus	2007	FJ573184
North Island	New Zealand	Iris	1-Sep-05	FJ618533
Lucknow	India	Gladiolus	15-Jan-11	JF682235
Bate9	Australia	Iris	1-Nov-10	JN127345
Glad-4	India	Gladiolus	Mar-11	JN692496
Glad-9	India	Gladiolus	Mar-11	JN692497
Glad-7	India	Gladiolus	Mar-11	JN692498
Glad-6	India	Gladiolus	Jan-12	JQ686720
Glad-8	India	Gladiolus	Jan-12	JQ686722
Glad-3	India	Gladiolus	Jan-12	JQ686723
SW3.1	Australia	Diuris	Aug-11	JQ807995
SW3.3	Australia	Diuris	Aug-11	JQ807996
KP	Australia	Diuris	Aug-11	JQ807997
Crocosmia	Netherlands	Crocosmia	Jun-11	KF493898
2677-NL	Netherlands	Crocus	23-Nov-13	KJ194467
Taean	South Korea	Gladiolus	23-Jun-11	KU981083

n/a, no data available
