# Supplementary material for: Genetic diversity and molecular evolution of Ornithogalum mosaic virus based on the coat protein gene sequence
Source: PeerJ. 2018 Mar 27;6:e4550. doi: 10.7717/peerj.4550 (PMC5877448; doi:10.7717/peerj.4550)
Supplement: Table S2 [file peerj-06-4550-s002.pdf]

Table S2 Recombination events detected in OrMV CP gene by RDP4 Suites

No.	Recombinant	Break point (nt)	Parent isolate (Major × Minor)	Methods with P-value ($< 10^{-4}$)*
1	Crocosmia	571-749	Glad-6 × Unknown	G, B, M, C, S, 3S
2	Glad-7	407-751	Glad-4 (Glad-9) × Unknown	G, M, S, 3S
3	OMV-O, 7-3, OMV-IL, nzOrMV-1, nzOrMV-2	747-172	G × Lucknow	R, M, C, 3S
4	Glad-8	256-333	Glad-6 × Glad-3	G, M, S, 3S

*R, RDP; G, Geneconv; B, BootScan; M, Maxchi; C, Chimarera; S, SiScan; 3S, 3Seq
