# Supplementary material for: Genetic diversity and molecular evolution of Ornithogalum mosaic virus based on the coat protein gene sequence
Source: PeerJ. 2018 Mar 27;6:e4550. doi: 10.7717/peerj.4550 (PMC5877448; doi:10.7717/peerj.4550)
Supplement: Table S3 [file peerj-06-4550-s003.pdf]

Table S3 (a) Genetic diversity parameters estimated for the CP gene of OrMV. (b) Summary of test statistics for population differentiation

**Phylogenetic Group**	**Sample size**	**Haplotypes**	**Haplotype diversity**	**Nucleotide diversity**
(a)				
Group 1	23	23	1.000 (±0.013)	0.106 (±0.014)
Group 2	13	13	1.000 (±0.030)	0.117(±0.015)
Total	36	30	1.000 (±0.007)	0.156 (±0.010)

**Phylogenetic Group**	K_{ST}	K_S	***P-value***	S_{nn}	***P-value***	F_{ST}	***P-value***
(b)							
Group 1 vs. Group 2	0.296	83.545	< 0.001***	1.00	< 0.001***	0.470	< 0.001***

Significance thresholds: *, 0.01< *P-value*<0.05; **, 0.001< *P-value*<0.01; ***, *P-value*<0.001
