# Supplementary material for: Genetic diversity and molecular evolution of Ornithogalum mosaic virus based on the coat protein gene sequence
Source: PeerJ. 2018 Mar 27;6:e4550. doi: 10.7717/peerj.4550 (PMC5877448; doi:10.7717/peerj.4550)
Supplement: Table S4 [file peerj-06-4550-s004.pdf]

Table S4 (a) Results of CODEML analyses of selective constraint for CP gene of OrMV. (b) Codon positions under positive selection

OrMV group	Model	Parameter estimates (Frequency, *f* and ω Values)	x^2	Ln L	LRT *p*-value
(a)					
Clade A	One-ratio 0 (ω_0=ω_1)	ω_0=ω_1=0.152	7.332	-6778.939	<0.001
	Two-ratio 2 (ω_0, ω_1)	ω_0=0.159, ω_1=0.055		-6775.272	
Clade B	One-ratio 0 (ω_0=ω_1)	ω_0=ω_1=0.152	8.437	-6778.939	<0.001
	Two-ratio 2 (ω_0, ω_1)	ω_0=0.159, ω_1=0.028		-6774.720	

OrMV Group	Model (np)	Parameter estimates	Positive sites	Ln L	LRT *p*-value
(b)					
All	M3 (74)	*p*: 0.558, 0.442, 0.000	n/a	-6115.413	<0.001
		ω: 0.050, 0.330, 46.913			
	M0 (70)	ω_0: 0.161	Not Allowed	-6169.647	
	M2a (73)	*p*: 0.926, 0.047, 0.028	n/a	-6154.614	1.000
		ω: 0.143, 1.000, 1.000			
	M1a (71)	*p*: 0.926, 0.074,	Not Allowed	-6154.614	
		ω: 0.143, 1.000,			
	M8 (73)	p_0=0.999, *p*=0.728, *q*=3.136 (p_1= 0.00001), ω= 1.000	n/a	-6116.037	0.999
	M7 (71)	*p*= 0.723, *q*=3.136	Not Allowed	-6116.036	
Clade A	M3 (48)	*p*: 0.513, 0.487, 0.000	n/a	-4289.288	<0.001
		ω: 0.080, 0.464, 34.976			
	M0 (44)	ω_0: 0.251	Not Allowed	-4311.891	
	M2a (47)	*p*: 0.865 0.084, 0.050	n/a	-4298.504	1.000
		ω: 0.195, 1.000, 1.000			
	M1a (45)	*p*: 0.865, 0.135	Not Allowed	-4298.504	
		ω: 0.195, 1.000			
	M8 (47)	p_0=0.999, *p*=0.799, *q*=2.060 (p_1= 0.00001), ω= 1.000	n/a	-4289.356	0.999

	M7 (45)	p=0.799, q=2.060	Not Allowed	-4289.356	
Clade B	M3 (30)	p: 0.807, 0.193, 0.000	n/a	-2470.915	<0.001
		ω: 0.022, 0.317, 36.191			
	M0 (26)	ω_0: 0.072	Not Allowed	-2488.257	
	M2a (29)	p: 0.955, 0.03314, 0.012	n/a	-2476.363	1.000
		ω: 0.052, 1.000, 1.000			
	M1a (27)	p: 0.955, 0.045	Not Allowed	-2476.363	
		ω: 0.052, 1.000			
	M8 (29)	p_0=0.993, p=0.196, q=1.883 (p_1=0.007), ω=1.694	15S, 30S, 36Q	-2469.270	0.107
	M7 (27)	p=0.176, q=1.494	Not Allowed	-2471.507	
