# Supplementary figures and images for: Genetic diversity and molecular evolution of Ornithogalum mosaic virus based on the coat protein gene sequence
Source: PeerJ. 2018 Mar 27;6:e4550. doi: 10.7717/peerj.4550 (PMC5877448; doi:10.7717/peerj.4550)

Figure S1

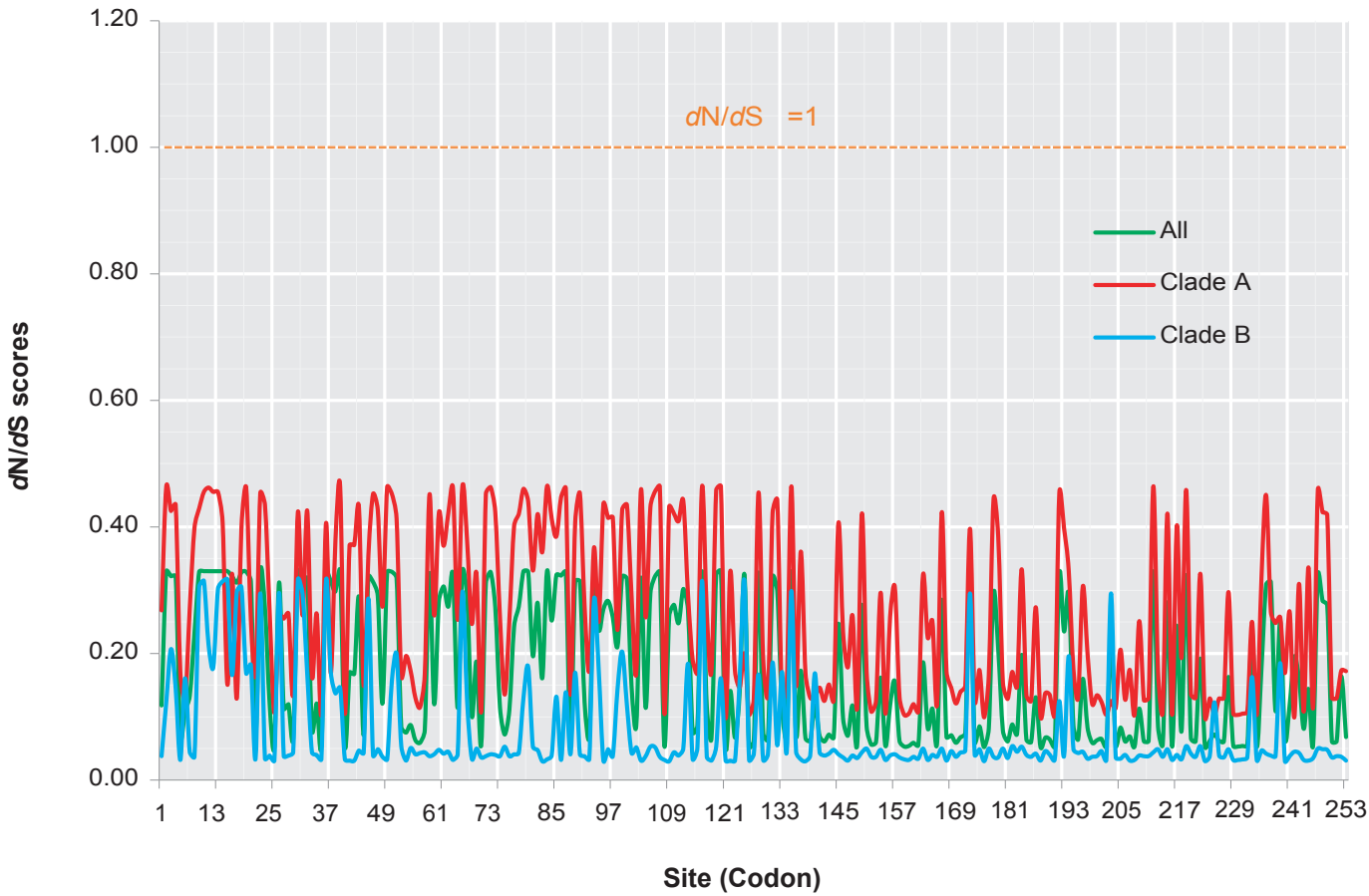

Supplement: Figure S1 — Sites under neutral (dN/dS = 1) are marked in orange dotted line and each phylogenic clade is indicated with a unique color. The window size is 13 codons, and the offset between windows is one codon. [file peerj-06-4550-s005.pdf]
